# Experimental Parametric Investigation of Nanosecond Laser-Induced Surface Graphitization of Nano-Crystalline Diamond

**DOI:** 10.3390/ma17112704

**Published:** 2024-06-03

**Authors:** Huixin Yuan, Liang Zhao, Junjie Zhang

**Affiliations:** 1 Center for Precision Engineering, Harbin Institute of Technology, Harbin 150001, China; hithuixin@163.com; 2 Shenyang Aircraft Industry (Group) Co., Ltd., Shenyang 110850, China; zhaoliang.0403@gmail.com

**Keywords:** nano-crystalline diamond, laser ablation, graphitization, Raman spectroscopy, surface morphology

## Abstract

While nano-crystalline diamond (NCD) is a promising engineering composite material for its unique mechanical properties, achieving the ultrahigh surface quality of NCD-based components through conventional grinding and polishing is challenging due to its exceptional hardness and brittleness. In the present work, we experimentally investigate the nanosecond laser ablation-induced graphitization characteristics of NCD, which provides a critical pretreatment method of NCD for realizing its superlative surface finish. Specifically, systematic experimental investigations of the nanosecond pulsed laser ablation of NCD are carried out, in which the characteristics of graphitization are qualitatively characterized by the Raman spectroscopy detection of the ablated area of the microhole and microgroove. Subsequently, the influence of laser processing parameters on the degree and morphological characteristics of graphitization is evaluated based on experimental data and related interpretation, from which optimized parameters for maximizing the graphitization of NCD are then identified. The findings reported in the current work provide guidance for promoting the machinability of NCD via laser irradiation-induced surface modification.

## 1. Introduction

The composite material of nano-crystalline diamond (NCD) is extensively employed for the fabrication of high-precision cutting tools, optical components, and precision instruments due to its exceptional hardness, superior thermal stability, and high wear resistance [1,2,3,4,5]. Those applications of NCD-based components exceedingly demand high surface integrity and low surface roughness. However, the ultralow machinability accompanied by the intrinsic hardness and brittleness of NCD presents significant challenges for its traditional grinding, polishing, and chemical-mechanical polishing processes [6,7,8,9,10,11]. Thus, prompting the machinability of NCD is highly desirable.

The transformation of diamond into graphite, which are the two most prevalent allotropes of carbon, is a widely observed phenomenon of graphitization under high temperatures or high pressures. Since graphite exhibits significantly lower hardness than diamond, the graphitization of diamond results in a substantial decrease in hardness and an enhancement in ductility, which are advantageous for the subsequent grinding and polishing processes of diamond. In particular, the laser-induced graphitization of single-crystal diamond has been widely reported [12,13]. Specifically, laser irradiation-induced temperatures higher than approximately 700 °C result in the rearrangement of carbon atoms from sp^3^ hybridized diamond to sp^2^ hybridized graphite, the formation of which significantly enhances the subsequent absorption of deposited laser power.

Nanosecond pulsed laser ablation, which is recognized for its high-peak power and short interaction time, has been extensively utilized for achieving localized thermal effects, facilitating the thermal-induced graphitization of diamond. As compared to femtosecond lasers and picosecond lasers, a nanosecond laser has longer pulse widths and higher ablation rates, which makes diamond easier to be graphitized [12,14,15,16]. We also note that the ultrashort pulsed laser with a reduced thermal effect and effective propensity of periodic surface nanoscale structures is beneficial for accurately diamond microstructuring [17]. Zhang et al. reported two typical altered layers of graphite formed in the infrared nanosecond laser ablation of single-crystal diamond, as an induced altered layer and a deposited altered layer. While the induced altered layer serves as a transitional phase from diamond to existing graphite phases, the deposited altered layer, comprising graphite and amorphous carbon deposited on the microgroove surface, forms through the nucleation and deposition of carbon islands [18]. Ohfuji et al. utilized a ultraviolet nanosecond laser to fabricate microgrooves on single-crystal diamond surfaces, resulting in the formation of a graphite layer composed of single-crystal, polycrystalline, and nanocrystalline graphite [19]. Furthermore, Zhao et al. investigated the evolution of the microstructures in laser-processed single-crystal diamond surfaces, which reveals the presence of graphite and amorphous carbonate layers [20]. Therefore, the nanosecond laser-induced graphitization of NCD is a promising pretreatment method for prompting its machinability.

Although the nanosecond laser-induced graphitization of single-crystal diamond is well documented, the laser ablation-induced phase transformation in NCD is largely unknown. NCD, characterized by its nanoscale grains and a high density of grain boundaries (GBs), exhibits graphitization behavior that is distinctly different from that of the single-crystal counterpart. In particular, NCD exhibits a strong tendency toward graphitization in the GB regions due to the disordered atom nucleation in these regions that facilitates the transition from diamond to graphite even at low temperatures. Consequently, the abundance of GBs in NCD complicates the graphitization process from that of single-crystal diamond. Therefore, there is an urgent need for the in-depth understanding of the nanosecond laser ablation process of NCD, especially its unique characteristics of graphitization. Okuchi et al. fabricated microgrooves on a NCD surface using a near-UV nanosecond laser [21]. They found that the groove surface was covered by a graphite layer with a thickness of approximately 1 μm, and the diamond–graphite interface was almost linear, with a surface roughness less than 100 nm. However, the groove depth obtained in this experiment was on the order of hundreds of micrometers, which is too large for the subsequent polishing process. Currently, there is a lack of studies on the morphology and graphitization of NCD under laser ablation processes accompanied by a few micrometers of material removal.

The degree and uniformity of graphitization in the laser ablation of diamond are strongly dependent on various laser processing parameters, such as the average laser power, laser pulse width, scanning rate, and defocusing amount. The characteristics of graphitization are influenced by the energy density distribution and the overlapping rate of the adjacent laser spots, both of which are determined by these parameters. Kononenko et al. experimentally studied the effect of the laser pulse width on the graphitization of polycrystalline diamond [22]. Their results showed that the larger the laser pulse width, the thicker the graphite layer formed on the diamond surface, and the higher the ablation rate. Wu et al. used a nanosecond laser to fabricate microgroove arrays on the single-crystal diamond surface, and investigated the effects of the laser power and scanning rate on the microgroove depth, material removal rate, and surface topography [23]. They found that the material removal rate and microgroove depth both increased with the laser power. The microgroove depth shows a nonlinear decreasing trend with the increasing scan speed, while the material removal rate shows the opposite trend. While the influence of the laser processing parameters on the laser-induced graphitization of single-crystal diamond has been extensively studied, the patterns and mechanisms of NCD graphitization under different laser processing parameters have not yet been reported. The dominant thermal effect in the nanosecond laser leads to the extensive heating of the ablated surface area due to heat diffusion, resulting in a widespread and uniform surface graphitization, which is beneficial for realizing the extensive and uniform surface graphite transformation. In contrast, the femtosecond laser processing normally leads to the localized and non-uniform phase transformation of diamond due to the significantly reduced thermal effect. Specifically, femtosecond laser processing is driven by non-thermal effects such as multiphoton absorption and electron–lattice interactions, which results in a significant concentration of energy on the surface, the generation of high-density transient electronic excitation, and the formation of a thinner and non-uniform graphitized layer [17,24,25].

While graphitization is promising in promoting the machinability of NCD, performing laser processing parameter optimization is crucial for maximizing the graphitization that corresponds to the most pronounced machinability of NCD. Therefore, in the present work, we perform the experimental investigation of the nanosecond laser ablation of NCD, with an emphasis on the effect of the laser processing parameters on the ablated surface morphology and characteristics of graphitization. Specifically, systematic single-variable controlled experiments of the nanosecond laser ablation of NCD are performed to determine the impact of each laser processing parameter on the formation of the graphite layer. Subsequently, the optimal parameters for the most pronounced laser-induced graphitization of NCD materials is identified.

## 2. Materials and Methods

In the present study, the utilized bulk NCD sample prepared via chemical vapor deposition has dimensions of 2 mm × 2 mm × 1 mm. The NCD has a grain size in the range of 50–100 nm, a density of 3.52 g/cm³, and a hardness of 99 GPa. The details about the properties of the NCD can be found elsewhere [26].

**Figure 1:**
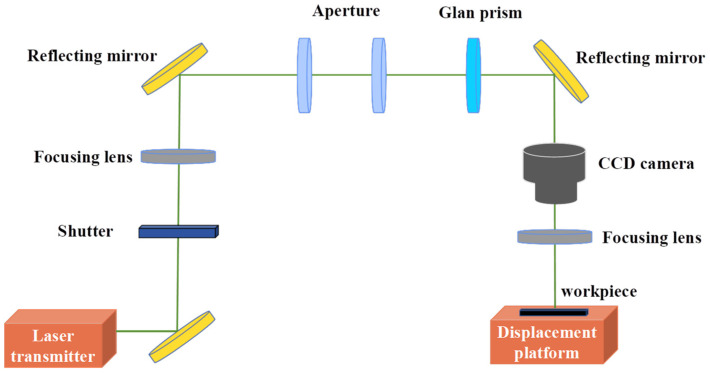
Illustration of the experimental configuration of nanosecond pulse laser ablation.

Figure 1 illustrates the experimental setup of the nanosecond laser ablation of NCD, which contains various types of lenses, laser sources, CCD microscope systems, and sample stages. The utilized laser source is a nanosecond pulse YAG laser with a wavelength of 532 nm, a laser spot diameter of 10 µm, a pulse width of 5 ns, and a pulse repetition rate ranging from 500 to 20,000 Hz. The laser beam profile can be shaped via employing a diaphragm to trim its irregular edges, with the diameter of the diaphragm being approximately 10 mm. Additionally, a granular prism enables the precise adjustment of laser power with an accuracy of 0.1 mW. The in situ observation of the processing surface is facilitated by a CCD camera, providing essential information concerning the focal point calibration. The laser ablation experiment is conducted in a constant temperature and dust-free workshop environment at 20 °C, and the laser ablation time is 100 ms.

The morphology of the ablated surface of NCD is characterized using a scanning electron microscope (SEM). Since NCD is a non-conductive material, the SEM characterization experiment is performed through depositing a layer of Au film with a thickness of about 5 nm on the surface of NCD samples, in such a way that the conductivity is effectively improved while the microstructure characteristics are retained. Furthermore, the three-dimensional optical profilometer is employed to measure the 3D surface morphology with a cross-sectional profile of the ablated NCD surface.

The laser-induced graphitization of NCD is characterized using a Raman Spectrometer, which serves as a crucial tool for characterizing the structural and chemical properties of diamond. Raman spectroscopy detection is carried out using a 532 nm wavelength for the diamond phase, which has a high scattering cross-section for the diamond phase to effectively excite the vibration mode of carbon atoms in the diamond. Consequently, the efficiency of maximizing Raman signals is achieved, accompanied by the minimization of possible photo-induced damage or material structure changes. The peak of Raman spectra for the sp^3^ hybridization bonded diamond typically manifests at approximately 1332 cm^−1^, which is attributable to the first-order scattering of vibration modes from carbon atoms within diamond crystals. Conversely, the Raman spectra peak of graphitized diamond shifts to the G peak and D peak. The G peak is situated around 1580 cm^−1^, which is associated with the characteristic Raman scattering signal produced by sp^2^ hybridized carbon atoms in graphite. The D peak, on the other hand, is located at roughly 1350 cm^−1^, which is linked to an irregular vibration mode, stemming from either defective graphite structures or disordered carbon materials containing sp^2^ hybridized carbon atoms. A higher degree of graphitization is correlated with a larger I_G_/I_D_ ratio. This is because, within this ratio, ‘I_G_’ denotes the peak intensity of graphitized carbon, while ‘I_D_’ represents the peak intensity of amorphous carbon. An increase in the I_G_/I_D_ ratio signifies a decrease in the content of amorphous carbon relative to graphitized carbon, thereby indicating an enhancement in the degree of graphitization.

## 3. Results and Discussion

### 3.1. Characteristics of Laser-Induced Graphitization of NCD

**Figure 2:**
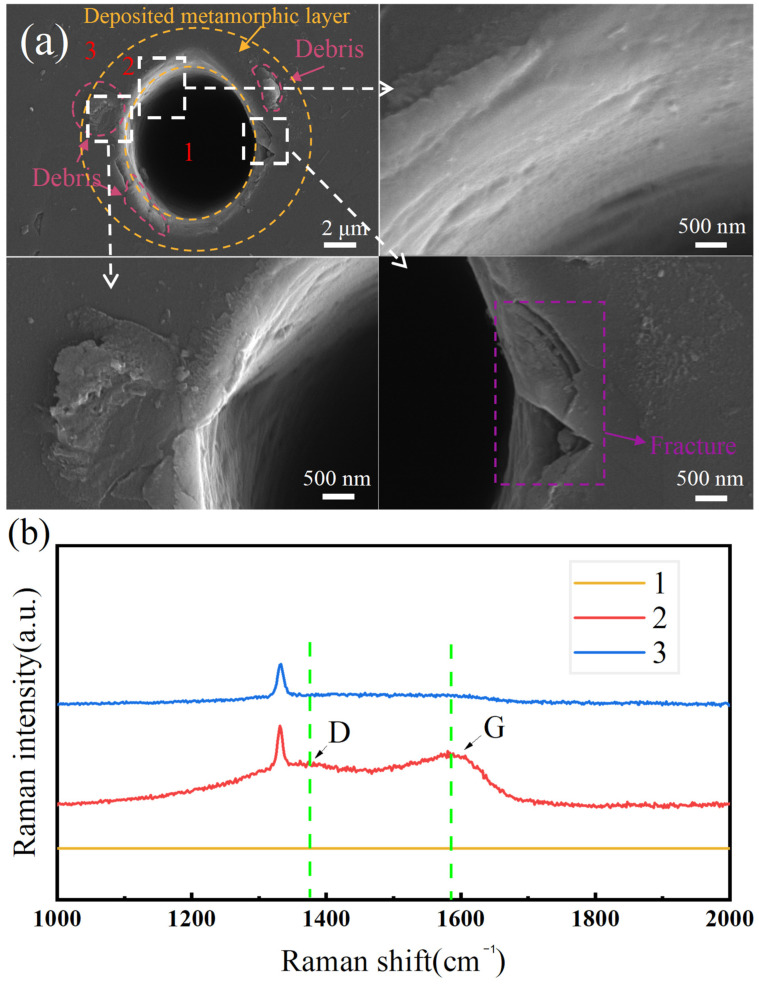
(**a**) Typical microhole morphology and (**b**) Raman spectra of the laser-irradiated NCD surface.

The nanosecond laser ablation of NCD in the point scanning mode, under a laser power of 25 mW and a repetition rate of 1500 Hz, is firstly carried out to reveal the characteristics of graphitization. Region 1 represents the central position of the hole, while region 2 denotes the edge position of the hole. Region 3, on the other hand, refers to the diamond substrate material. Figure 2a presents SEM images of the ablated NCD surface, which indicates that laser point scanning induces substantial microstructural evolutions in the NCD. Figure 2a demonstrates the formation of a microhole, accompanied by the evaporation of materials, which is due to the higher laser power used above the ablation threshold of the material. The ablated diamond material displays a characteristic of surface ablation morphology under the nanosecond pulsed laser exposure, accompanied by the visible accumulation of fragmented material in the vicinity of the hole walls. While the hole edge remained relatively intact, the microcracks propagate outward to induce pronounced chipping. This phenomenon can be attributed to the compressive stress exerted on the diamond surface at high laser power densities. Furthermore, subsequent graphitization accompanied by the volume expansion of the graphitized region induces additional compressive stress, ultimately leading to the formation of surface cracks. Concurrently, a noticeable ring-shaped material deposition with a distance of approximately 1 μm from the hole edge is observed.

The hard and brittle nature of diamond results in the formation of evident brittle cracks in the lower right corner of the ablated NCD surface. The annotations in Figure 2a shows the laser ablation-induced fracture and debris structures, which indicate the changes in the material’s surface topography and composition. Specifically, the term ‘deposited metamorphic layer’ refers to a layer of diamond that has undergone structural transformation due to the laser ablation-induced thermal effect. The term ‘fracture’ indicates where cracks are developed in the diamond, likely due to the thermal stress or the high pressure impact of the laser beam. The term ‘debris’ refers to the Raman spectroscopy results of the inner hole, the deposition area, and the diamond surface away from the laser-affected zone, revealing the apparent presence of graphite phases on the hole walls. Figure 2b presents the Raman spectroscopy results from three distinct regions, as region 1 represents the microhole region, region 2 denotes the material deposition area surrounding the diamond microhole, and region 3 signifies the diamond substrate. It is seen from Figure 2b that the microhole region does not yield any valid data for the Raman spectrometer, most likely because the depth of the processed microholes is too large to be detected. The presence of a D peak and a G peak in the deposition area suggests the occurrence of graphitization within this region. This phenomenon is attributed to the phase transformation from diamond to graphite on the laser ablated surface. Furthermore, only one characteristic diamond peak is discerned in the Raman spectrum of the diamond substrate.

The nanosecond laser ablation of NCD leads to the formation of a metamorphic layer at the edge of the microhole, which is attributed to the inherent high thermal gradient and rapid cooling effects in the laser ablation process. The observation of graphitization at the hole edge suggests a phase transformation caused by localized temperatures exceeding the stability range of the diamond, resulting in a change in the crystalline structure from diamond to graphite. Since the laser ablation-induced brittle cracks on the NCD surface further develop to cause severe surface damage during the subsequent grinding process, it is necessary to rationally select the laser processing parameters in order to maximize graphitization while minimizing the generation of brittle surface cracks, thus achieving high surface integrity.

### 3.2. Effect of the Pulse Repetition Rate on the Graphitization of NCD

**Figure 3:**
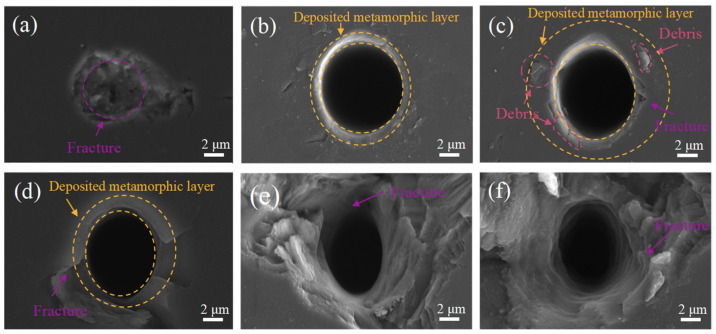
SEM images of the ablated NCD surface under pulse repetition rate of (**a**) 500 Hz, (**b**) 1000 Hz, (**c**) 1500 Hz, (**d**) 2000 Hz, (**e**) 5000 Hz, and (**f**) 10,000 Hz.

The nanosecond laser ablation of NCD is performed to investigate the influence of the pulse repetition rate on the graphitization of NCD. Six pulse repetition rates, namely 500 Hz, 1000 Hz, 1500 Hz, 2000 Hz, 5000 Hz, and 10,000 Hz, are considered. For each pulse repetition rate, the other laser processing parameters are the same, with a laser power of 25 mw and a laser ablation time maintained at 100 ms. Figure 3 shows SEM images of the ablated surface of NCD under different pulse repetition rates. It is seen from Figure 3 that the pulse repetition rate has important effects on the hole morphology. Figure 3a shows that, when the pulse repetition rate is 500 Hz, the diamond surface is only slightly fractured, without the formation of microholes. When the pulse repetition rate exceeds 1000 Hz, the pronounced formation of microholes is evident on the ablated diamond surface, as illustrated in Figure 3b–f. Figure 3b shows that, at a laser repetition rate of 1000 Hz, the hole structure is relatively intact, with no cracks observed at the periphery. However, as depicted in Figure 3c, at a repetition rate of 1500 Hz, microcracks emerge at the hole edge and propagate outwards, accompanied by conspicuous spalling. This phenomenon can be attributed to the generation of substantial compressive stress on the diamond surface at elevated repetition frequencies, which is accompanied by volume expansion within the graphitization area, subsequently leading to surface cracking. As demonstrated in Figure 3c–f, between laser repetition frequencies of 2000 and 10,000 Hz, there are significant fragmentations formed on the ablated diamond surface, with pronounced brittle fractures appearing along the hole sidewall. The fracture structures on the surface of NCD deteriorate the machined surface quality during subsequent grinding processes. Consequently, a pulse repetition rate of 1000 Hz is recommended for achieving the optimal hole surface quality through laser processing.

**Figure 4:**
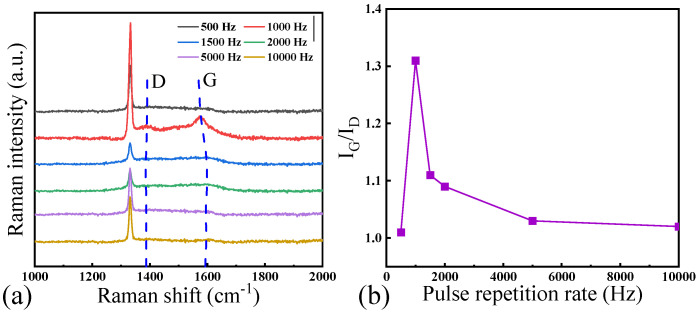
(**a**) Raman spectra and (**b**) I_G_/I_D_ ratio at hole edge on laser ablated NCD surfaces under different pulse repetition rates.

Figure 4a shows the results of the graphitization of NCD under different pulse repetition rates characterized by Raman Spectrometer. Figure 4b further shows the IG/ID ratio derived from the Raman spectrometry characterization. When the pulse repetition rate is 500 Hz, no graphitization has occurred on the diamond surface. The analysis results reveal that, under pulse repetition rates between 1000 Hz and 1500 Hz, the sidewalls of the holes undergo graphitization. When the pulse repetition rate is higher than 2000 Hz, the fractured structure at the hole edge on the NCD surface is mainly diamond, and there is no graphite observed. The comprehensive analysis shows that, when the pulse repetition rate is 1000 Hz, the NCD surface integrity is the best, and the sidewall is obviously graphitized. Further analysis reveals a strong correlation between the degree of laser ablation-induced graphitization of NCD and the topography of the ablated surface. When the diamond surface exhibits complete hole topography, an increased degree of graphitization is observed. Conversely, when the diamond is increasingly fractured, the degree of graphitization decreases. Consequently, optimal laser ablation-induced graphitization occurs at a pulse repetition rate of 1000 Hz.

### 3.3. Effect of Laser Power on the Graphitization of NCD

**Figure 5:**
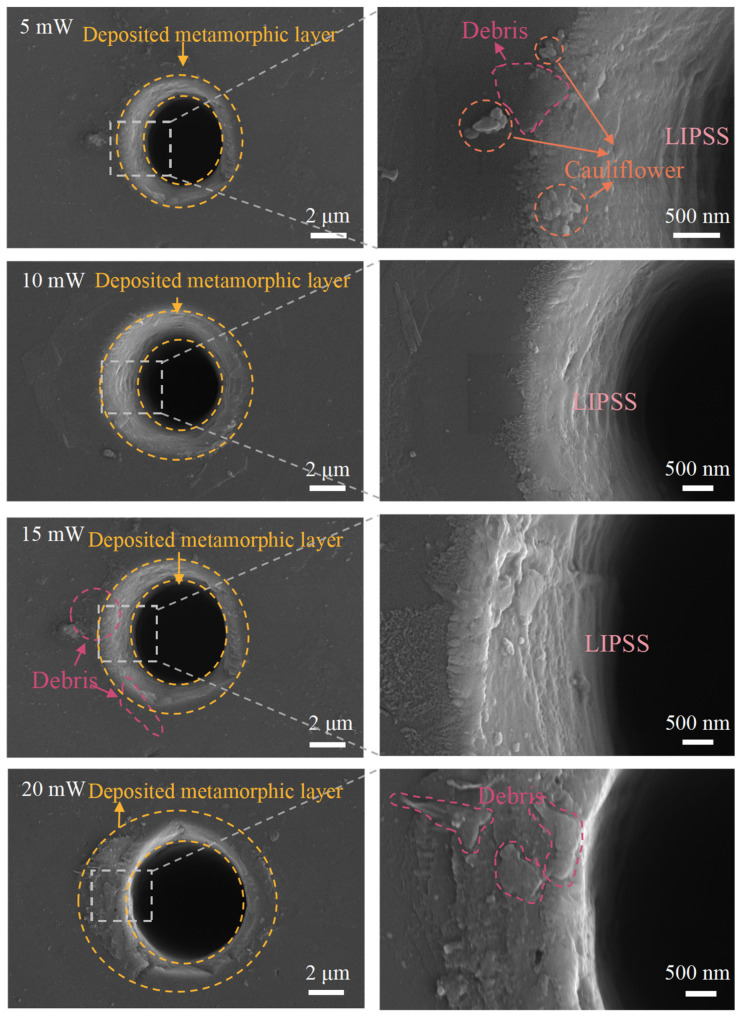

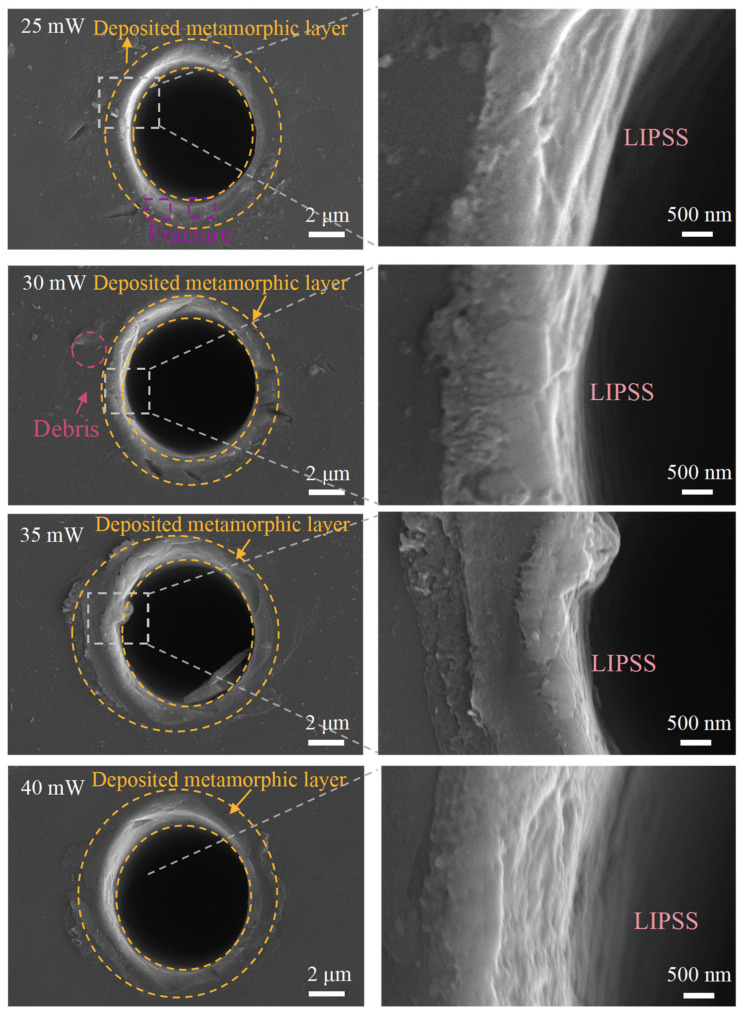
Influence of laser power on the morphology of ablated NCD surface.

With the optimal laser pulse frequency of 1000 Hz, the subsequent nanosecond laser ablation of NCD is carried out to investigate the effect of laser power on the graphitization of NCD. The laser power ranges from 5 mw to 40 mw with an interval of 5 mw. For each laser power, the other laser processing parameters are the same, at a laser pulse frequency of 1000 Hz and a laser ablation time of 100 ms. Figure 5 illustrates the ablated surface morphology of NCD at varying laser powers. These morphologies can be categorized into three primary regions, as microholes, deposited zones, and the diamond matrix, respectively. The laser power exerts a significant influence on both the microholes and the obtained surface integrity. Upon examining the hole size, it is observed that an increase in laser power leads to a corresponding enlargement of the microhole diameter. Additionally, a comprehensive analysis of the laser ablated area reveals that, at laser power levels ranging from 5 to 20 mW, the sidewalls of microholes exhibit a pronounced textured structure. However, when the laser power exceeds 25 mW, a higher laser power results in a more prominent deposition layer on these sidewalls. Moreover, when the laser power is higher than 35 mW, fragmentation and fracture structures are observed along the edges of microholes. While a high laser power facilitates the deposition layer formation, it is prone to induce surface collapse and edge cracks. A comparative analysis demonstrates that, at laser powers of 25 mW and 30 mW, the ablated NCD surfaces exhibited maximum completeness with the most pronounced formation of deposited layers.

In addition, the sidewall of the hole also exhibits the formation of laser-induced periodic surface structures (LIPSSs). The formation of LIPSSs is a result of the intricate interaction between lasers and materials, encompassing electromagnetic, thermal, and material properties. The interaction between lasers and materials involves a localized enhancement effect of the electromagnetic field when a laser beam irradiates the surface of diamond. This effect leads to interference between surface electromagnetic waves and the incident laser, resulting in the periodic distribution of electromagnetic fields. Additionally, high-energy laser-induced surface plasmon waves propagate at the interface of diamond, causing local melting and recrystallization on the material’s surface to form periodic nanostructures. Furthermore, transient thermal gradients caused by laser irradiation lead to thermo-mechanical effects such as thermal stress and expansion effects, inducing physical deformation between heated and non-heated areas of the material, which results in the formation of periodic surface ripples. Lastly, due to its high anisotropy, differences in absorption and reflection behaviors on different crystal faces during laser energy deposition further promote the formation of periodic structures in diamond [17,27,28].

**Figure 6:**
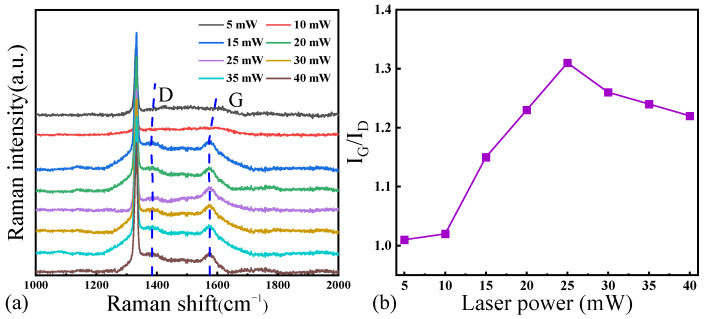
(**a**) Raman spectra and (**b**) I_G_/I_D_ ratio of the inner surface of NCD at different laser powers.

Figure 6a shows the results of Raman spectroscopy on the surface of the laser-processed hole at different laser power levels, and Figure 6b shows the corresponding I_G_/I_D_ ratio in the Raman spectroscopy curve. The observations of the G peak and the D peak indicate varying degrees of graphitization on the NCD surface during laser processing. Initially, there is an increase in the degree of graphitization on the NCD surface, followed by a subsequent decrease, ultimately reaching its maximum at a laser power of 25 mW. Achieving a significant phase transition towards graphitization at a low laser power proves to be challenging, while an excessively high laser power leads to a reduction in the degree of graphitization on the diamond surface. Furthermore, the degree of the graphitization of the diamond surface is correlated with its surface topography; the distribution of diamond deposits at a laser power of 25 mW exhibits the highest level of uniformity. We also note that the graphitization is also strongly correlated with the distance from the laser spot, i.e., the center of the hole, despite the laser power.

**Figure 7:**
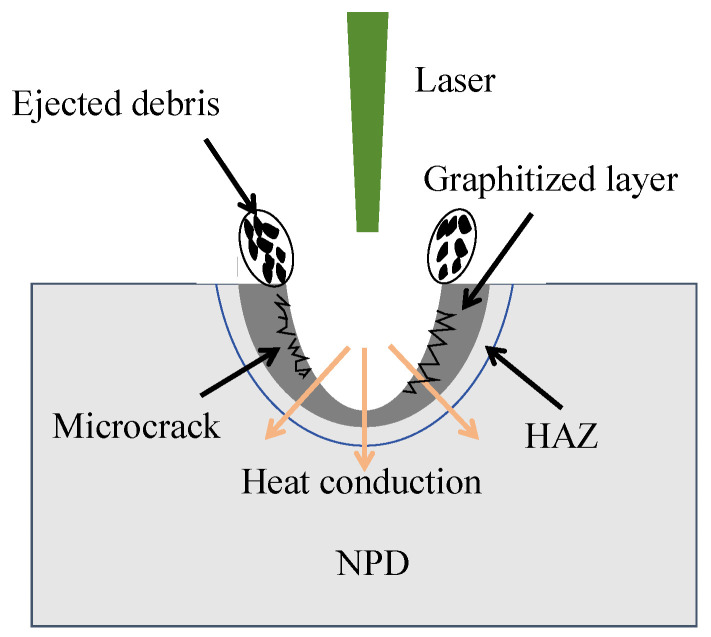
Schematic diagram of graphitization in the nanosecond laser ablation process of NCD.

Figure 7 illustrates the mechanism of nanosecond laser ablation in NCD. The interaction between the nanosecond laser and NCD induces the following two primary mechanisms: phase transformation and thermal stress. Initially, the high local energy density of the nanosecond laser leads to a significant energy transfer process. This sudden surge of energy triggers a phase transition within the diamond structure, typically resulting in a shift from the diamond phase to the graphite phase. Such a transition often manifests under a conversion from diamond to graphite or other carbon-based amorphous structures. This phase transformations encompasses both the absorption and release of power, as well as the restructuring of the crystal lattice. The intense concentration of laser power subsequently causes a rapid elevation of temperature on the ablated diamond surface, establishing a substantial temperature gradient between the surface and interior. The thermal gradient induces significant thermal stress, particularly impacting the microstructure of the material. It is possible that these thermal stresses will initiate microcracks within the diamond. The excellent thermal conductivity of diamond material (up to 2200 W/(m·K)) results in a significant temperature gradient effect during laser ablation. When compared to traditional ceramic materials such as aluminum oxide, which has a thermal conductivity of about 30 W/(m·K), diamond can more effectively conduct heat to distant areas, thereby reducing local overheating. This characteristic is particularly important in nanosecond laser ablation processes, as it helps reduce the heat-affected zone and the likelihood of thermal stress and thermal cracking [29].

The objective of laser pretreatment in the laser-assisted grinding of diamond is to develop a graphite layer with enhanced surface integrity, so this process involves minimizing material removal from the surface while maximizing the degree of graphitization. Furthermore, achieving this objective is crucial for enhancing both the surface quality and properties of the diamond material during laser-assisted grinding.

**Figure 8:**
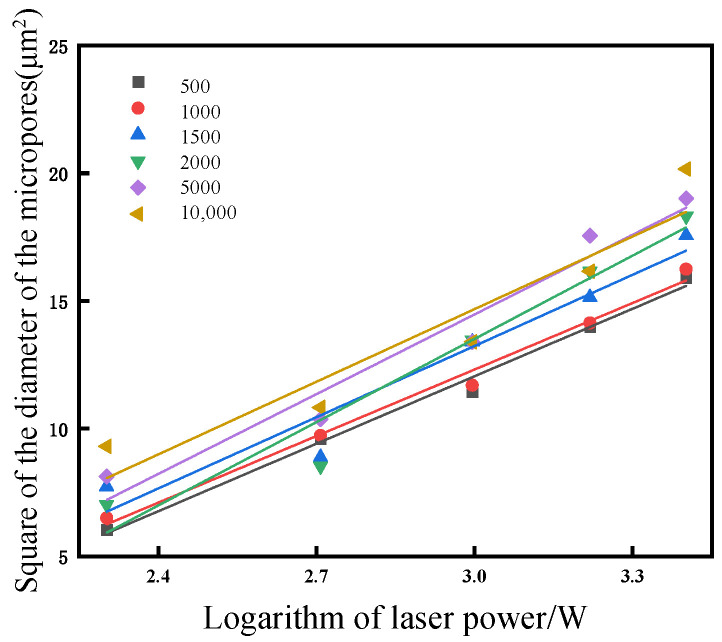
Relationship between the logarithm of laser power and the square of the diameter of the microholes.

In order to accurately determine the ablation threshold of diamond materials during nanosecond laser ablation processing, the diameters of the microholes formed on the diamond surface at various laser powers was measured, as depicted in Figure 8. The plotted linear data demonstrate a clear correlation between the square of the microhole diameter and the logarithm of the laser power density within the range of explored laser powers. By conducting regression analysis on this relationship, an ablation threshold of approximately 3.3 J/cm² is determined for the NCD when utilizing a laser spot radius of 5.6 μm.

### 3.4. Microgroove on NCD under Optimized Parameters

**Figure 9:**
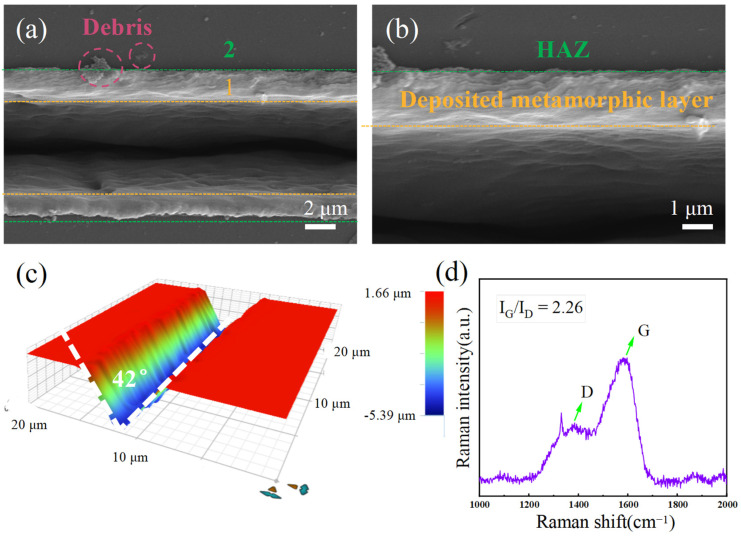
SEM image of (**a**) Overall surface morphology and (**b**) Sidewall surface morphology of laser-processed microgrooves on NCD surface; (**c**) Three-dimensional surface morphology of microgrooves on NCD; (**d**) Raman spectrum of the microgroove surface.

Nanosecond pulsed laser ablation experiments under the linear scanning mode are conducted to fabricate a microgroove on the NCD surface, employing the optimized laser parameters of a laser power of 25 mW, a repetition frequency of 1 kHz, and a scanning speed of 0.01 mm/s. Figure 9a–d shows the surface morphology of laser-processed NCD, the magnified view of the sidewall surface morphology of laser-processed microgrooves on the NCD surface, the three-dimensional surface morphology of laser-processed NCD, and the results of the Raman spectrum of the inner surface of NCD, respectively.

Figure 9a,b shows that the groove exhibits a V-shaped distribution, indicating the improved integrity of the lower surface, and evenly distributed sidewall textures without any brittle cracks. V-shaped microgrooves are observed on the diamond surface with evident material deposition on both sides and at the end where the laser beam terminates. However, it is noteworthy that the diamond surface adjacent to the groove appears smooth, suggesting minimal material deposition. The deposition morphology in region 1 is uniformly distributed with the absence of any debris particles. This can be attributed to the high plasma plume pressure, which facilitates material expansion into the surrounding atmosphere, and subsequently shields it with laser-induced plasma. Consequently, the deposition of debris particles is effectively prevented, establishing this area as the designated laser action deposition zone. The presence of debris particles in region 2 can be attributed to the gradual deposition of small splash particles onto the workpiece surface under the influence of gravity during processing. The deposition speed is higher for particles with larger volume and weight, resulting in their proximity to the laser action area. Conversely, smaller particles settle more slowly and deposit further away. The boundary between region 1 and region 2 is clearly distinguishable. Due to the energy distribution characteristics of the Gaussian beam, the central region of the microgroove exhibits maximum energy while it gradually decreases away from the groove. Therefore, area 2 is divided into a distinct heat-affected zone (HAZ). Figure 9c showed that the depth of the microgrooves is approximately 5 μm. The angle between the sidewalls of the grooves as 42° and the width of the microgrooves formed through machining is consistently uniform. The Raman spectroscopy plotted in Figure 9d was conducted on the central region of the groove, revealing the presence of the graphite phase. The obtained results indicate a G peak to D peak ratio of 1.342, suggesting graphitization within the groove region.

## 4. Conclusions

In summary, we experimentally investigate the mechanisms of surface graphitization, as well as its dependence on laser processing parameters, in the nanosecond laser ablation of NCD. The absorption of laser energy via NCD results in rapid thermal excitation, while elevated temperature and pressure lead to structural transformations, leading to the reconfiguration of carbon atoms into a graphite lattice. And the rationalization of laser parameters based on the surface morphology and graphitization degree of NCD suggests optimal values for the highest graphitization without any observable cracks or anomalies, at a laser power of 25 mW, a scanning speed of 0.1 mm/s, and a pulse repetition rate of 1000 Hz. The study demonstrates that, through the optimization of laser parameters, it is feasible to achieve a machined surface characterized by enhanced graphitization and minimal surface damage, which confers the advantages for promoting the machinability of NCD in subsequent grinding processes.

## Data Availability

The raw data supporting the conclusions of this article will be made available by the authors on request.
